# Clinical analysis of 19 patients with tuberculosis complicated by hemophagocytic lymphohistiocytosis

**DOI:** 10.3389/fimmu.2026.1781937

**Published:** 2026-03-13

**Authors:** Chun-Yan Zhao, Chang Song, Qing-Dong Zhu, Ai-Chun Huang, Tuo Lao, Xue-Wen Huang, Zhou-Hua Xie, Qiu-Qing Tan

**Affiliations:** 1 Department of Tuberculosis, The Fourth People’s Hospital of Nanning, Nanning, China; 2 Clinical Medical School, Guangxi Medical University, Nanning, China; 3 Department of Ophthalmology, People’s Hospital of Baise City, Baise, China

**Keywords:** clinical features, C-reactive protein, hemophagocytic lymphohistiocytosis, treatment, tuberculosis

## Abstract

**Background:**

To systematically describe the clinical characteristics of tuberculosis (TB) complicated by hemophagocytic lymphohistiocytosis (HLH), with the aim of informing early diagnosis, precise classification, and therapeutic decision-making.

**Methods:**

A total of 19 hospitalized patients diagnosed with TB-HLH between January 2020 and December 2025 were consecutively enrolled. Based on the presence of disseminated TB, patients were divided into a disseminated group (n = 10) and a non-disseminated group (n = 9). Demographic data, clinical symptoms, laboratory parameters, imaging findings, treatment regimens, and clinical outcomes were systematically collected and compared between the two groups.

**Results:**

No statistically significant differences were observed in baseline characteristics such as age and gender between the two groups (P > 0.05). Common clinical symptoms included fever, fatigue, weight loss, and hepatosplenomegaly. Central nervous system symptoms (e.g., headache, impaired consciousness) were observed only in the disseminated group (4 cases vs. 0 case, P = 0.033). The disseminated group had significantly lower serum C-reactive protein (CRP) levels compared to the non-disseminated group (73.20 ± 42.38 mg/L vs. 125.41 ± 61.45 mg/L, P = 0.044). Eighteen patients received anti-tuberculosis therapy, one patient with AIDS discontinued treatment due to critical illness. Glucocorticoids were administered to 16 patients as adjunct therapy for HLH. Among the three patients who did not receive steroids, one had a short disease course and mild bone marrow suppression, one presented with concurrent acute hepatic and renal failure, and one exhibited hematologic and symptomatic improvement following after anti-TB treatment. The overall mortality rate was 21.1% (4/19).

**Conclusion:**

This study suggests that central nervous system symptoms serve as a critical indicator for disseminated TB complicated by HLH. The inflammatory profile in TB-HLH exhibits unique features, such as relatively lower CRP in disseminated cases. Clinical management requires a combination of effective anti-TB therapy and a stratified immunomodulatory strategy based on the severity of the hyperinflammatory state.

## 1 Introduction

Tuberculosis (TB) remains a major global public health challenge. According to the latest World Health Organization (WHO) report, an estimated 10.7 million new TB cases occurred worldwide in 2024, with 1.23 million TB-related deaths, twice the number attributed to HIV/AIDS, maintaining TB’s position as the leading cause of death from a single infectious agent (1). In immunocompetent individuals, TB typically manifests as a localized infection with granuloma formation. However, when immune homeostasis is disrupted due to pathogen-related or host-related factors, it can escalate into a life-threatening systemic inflammatory response (2). Among the most severe manifestations of such immune dysregulation is hemophagocytic lymphohistiocytosis (HLH), also known as hemophagocytic syndrome (HPS). HLH is not a distinct disease but a critical clinical syndrome characterized by uncontrolled immune activation and a cytokine storm. Pathophysiologically, HLH is driven by impaired cytotoxic function of T cells and natural killer (NK) cells, leading to impaired antigen clearance and sustained immune activation. A Hyperactivated lymphocytes and macrophages produce excessive inflammatory cytokines, including interferon-gamma (IFN-γ), tumor necrosis factor-alpha (TNF-α), and interleukin-6 (IL-6), culminating in a cytokine storm (3). This hyperinflammation causes widespread tissue damage and leads to pathological macrophage activation in hematopoietic organs such as the bone marrow, liver, spleen, and lymph nodes, resulting in hemophagocytosis and cytopenias. Diagnosis of HLH relies on established clinical and laboratory criteria, such as those outlined in the HLH-2004 protocol. These include fever, splenomegaly, cytopenias, hypertriglyceridemia or hypofibrinogenemia, markedly elevated ferritin, and increased soluble CD25 levels (4). HLH can be classified as primary (genetic) or secondary (acquired), with the latter often triggered by infections, malignancies, or autoimmune diseases (5). Among infection-associated HLH cases in adults, approximately 50% are attributed to infectious etiologies, with TB accounting for 38% of these, and bacterial infections only 9% (6).

TB-associated HLH (TB-HLH) occupies a complex intersection between infectious disease and immunopathology. On one hand, active TB itself can present with fever, hepatosplenomegaly, pancytopenia, and elevated inflammatory markers, features that significantly overlap substantially with HLH criteria, often leading to underdiagnosis when these manifestations are attributed to TB alone. On the other hand, the systemic inflammation and multi-organ dysfunction seen in HLH can obscure the typical local features of TB, complicating etiological diagnosis and delaying targeted anti-TB therapy (6). This “dual-masking” effect complicates clinical recognition and contributes to poor outcomes. Although less common than EBV-HLH or malignancy-associated HLH, TB-HLH carries a high mortality rate, with some studies reporting case-fatality rates approaching 49% (7). Delayed diagnosis and late initiation of treatment are the principal contributors to poor prognosis (7). Effective management requires a delicate therapeutic balance: t prompt administration of potent anti-TB therapy to control the underlying infection, coupled with timely immunosuppressive or immunomodulatory therapy, such as glucocorticoids or etoposide, to mitigate the lethal cytokine storm. However, immunosuppression increases the risk of secondary infections, which can themselves be fatal and may be misinterpreted as HLH relapse (8). Currently, there is currently no consensus on standardized protocols or on determining the optimal therapeutic window and dosing strategies (9).

Given these challenges, this study aims to systematically describe the clinical features, laboratory findings, treatment pathways, and outcomes of TB-HLH patients. Our goal is to provide clinicians with a clearer diagnostic framework, improve early recognition, and offer evidence-based guidance for individualized and staged therapeutic strategies, ultimately contributing to improve clinical outcomes in this high-risk patient population.

## 2 Materials and methods

### 2.1 Diagnostic criteria

Given the significant overlap in clinical manifestations between TB and HLH, coupled with the high mortality rate of TB-HLH which necessitates early and accurate diagnosis, this study established strict and stratified diagnostic criteria to ensure the scientific rigor and reliability of the enrolled cases. The diagnosis of TB strictly followed the Diagnosis of Pulmonary Tuberculosis (WS 288-2017) standards issued by the Chinese Center for Disease Control and Prevention (10). Based on etiological test results, cases were classified into two categories: pathologically confirmed and clinically diagnosed. Cases were considered pathologically confirmed if they met any of the following criteria: (1) Mycobacterium tuberculosis (MTB) detected in respiratory specimens (e.g., sputum), bronchoalveolar lavage fluid (BALF), or lung tissue biopsy specimens via smear microscopy, culture, or molecular biological testing (e.g., Xpert MTB/RIF); or (2) MTB detected in sterile body fluids or tissue specimens. For pathogen-negative but clinically highly suspected cases, the establishment of a clinical diagnosis required a comprehensive assessment of epidemiological history, typical clinical manifestations (e.g., cough and expectoration for ≥2 weeks, hemoptysis, night sweats, fatigue, fever), and chest imaging features suggestive of active TB lesions, supported by at least one of the following pieces of evidence: (1) pathological examination (e.g., lymph node or liver biopsy) showing characteristic changes such as caseating granulomas; (2) moderate or strong positive tuberculin skin test (TST) or positive interferon-gamma release assay (IGRA); or (3) effective response to diagnostic anti-tuberculosis treatment, defined as significant alleviation of clinical symptoms and absorption of imaging lesions. The diagnostic criteria for concurrent HLH were primarily based on the Chinese Expert Consensus on the Diagnosis and Treatment of Hemophagocytic Syndrome (10) and the HLH-2004 guidelines revised by the Histiocyte Society (4). Considering the limited accessibility of certain specific immunological indicators (such as NK cell activity and sCD25) in routine clinical testing under the HLH-2004 criteria, as well as the retrospective nature of this study, it was stipulated that the diagnosis must meet at least five of the following eight indicators: (1) Fever: body temperature persistently exceeding 38.5°C for more than 7 days; (2) Splenomegaly; (3) Cytopenia (affecting two or three cell lineages): hemoglobin <90 g/L, platelet count <100×10^9^/L, neutrophil count <1.0×10^9^/L, excluding bone marrow hypoplasia; (4) Dyslipidemia and coagulation abnormalities: triglycerides >3.0 mmol/L or ≥3 standard deviations above the normal value for age, and/or fibrinogen <1.5 g/L or ≤3 standard deviations below the normal value for age; (5) Hemophagocytosis in tissue: hemophagocytes observed in pathological examinations of bone marrow, spleen, liver, or lymph nodes; (6) Significantly elevated ferritin: serum ferritin level ≥500 μg/L, which serves not only as a diagnostic indicator but its significant elevation often suggests a critical condition; (7) Decreased NK cell activity: reduced or absent activity; and (8) Elevated soluble CD25: abnormally elevated levels of soluble interleukin-2 receptor (sIL-2R). It should be specifically noted that for indicators (7) and (8) listed above, if testing was restricted by the patient’s condition or availability, a clinical diagnosis of TB-HLH could still be established for inclusion in this study, provided that at least five of the other six criteria were met (which must include HLH or elevated ferritin) and other causes of hemophagocytic syndrome (such as Epstein-Barr virus infection or malignancies) were clinically excluded.

### 2.2 Study population

This retrospective study included hospitalized patients diagnosed and treated at the Fourth People’s Hospital of Nanning between January 2020 and December 2025. Inclusion criteria were including age ≥18 years, and meeting diagnostic criteria for both TB and HLH. Exclusion criteria included the presence of other confirmed HLH triggers (e.g., active EBV infection, hematologic malignancies) or missing critical clinical data. A total of 19 patients meeting all three diagnostic criteria were included in the analysis. Based on the extent of MTB dissemination, patients were divided into two groups: (1) Disseminated TB group: Defined by radiological (e.g., CT, MRI) and/or bacteriological evidence of MTB infection involving at least one extrapulmonary organ or system (e.g., central nervous system (CNS), lymph nodes, bones, abdominal organs, bone marrow). (2) Non-disseminated TB group: Defined by MTB infection confined to the lungs and/or pleura, without evidence of extrapulmonary spread.

Clinical data were systematically collected from the hospital’s electronic medical records, including: (1) Demographic and baseline characteristics (e.g., age, gender, residence, lifestyle habits); (2) Detailed clinical symptoms and signs (e.g., fever, respiratory and digestive manifestations, lymphadenopathy, hepatosplenomegaly); (3) Laboratory findings (e.g., complete blood count, inflammatory markers, lymphocyte subsets, liver and kidney function, metabolic parameters); (4) Imaging results, particularly chest CT findings; (5) Treatment details and clinical outcomes (e.g., anti-TB and immunomodulatory regimens, adverse reactions, hospital stay duration, survival/mortality). All data were independently extracted and cross-verified by two researchers to ensure accuracy and consistency. The study protocol was approved by the Ethics Committee of the Fourth People’s Hospital of Nanning (Approval No.: [2025]52). As a retrospective study involving anonymized data without personal identifiers, the requirement for informed consent was waived.

### 2.3 Statistical analysis

Statistical analysis was performed using SPSS 22.0. Continuous variables are expressed as mean ± standard deviation (SD), and categorical variables are presented as frequencies percentages (%). For continuous variables, the assumption of homogeneity of variance was assessed. If variances were equal, intergroup comparisons were conducted using the independent samples t-test, otherwise, Welch’s t-test was applied. Differences in categorical variables were analyzed using McNemar’s chi-square test. A two-tailed P-value <0.05 was considered statistically significant.

## 3 Results

### 3.1 Baseline characteristics

A total of 19 patients were included in this study, 10 in the disseminated TB group and 9 in the non-disseminated TB group. As shown in **Table 1**, there were no statistically significant differences between the groups in age, gender, ethnicity, residence, personal history (smoking, alcohol consumption), or history of TB treatment (initial treatment vs. retreatment) (all P > 0.05), indicating comparability between groups. Specifically, the mean age was 54.20 ± 16.46 years in the disseminated group and 48.89 ± 17.42 years in the non-disseminated group (P = 0.504). No significant differences were found in the distribution of categorical variables such as gender and ethnicity.

**Table 1:** Comparison of baseline characteristics.

Basic information	Total (N=19)	Non-disseminated TB (N=9)	Disseminated TB (N=10)	Test statistic	P value
Age	51.68 ± 16.67	48.89 ± 17.42	54.20 ± 16.46	0.683	0.504
Sex					
Male	14	8	6	2.039	0.153
Female	5	1	4		
Ethnicity					
Han	14	8	6	2.039	0.153
Zhuang	5	1	4		
Place of residence					
Rural	15	6	9	1.552	0.213
Urban	4	3	1		
Personal history					
Smoking	7	4	3	0.425	0.515
Alcohol abuse	3	2	1	0.532	0.466
Tuberculosis treatment					
Newly treated	16	8	8	0.281	0.596
Previously treated	3	1	2		

### 3.2 Clinical symptoms

As shown in **Table 2**, most clinical symptoms showed no statistically significant differences in incidence between the two groups (all P-values > 0.05). Fever was the most common symptom (18 cases), with a similar distribution across groups. Cough and sputum production were also prevalent (12 cases each). However, the presence of CNS symptoms, such as headache or altered consciousness, were observed exclusively in the disseminated TB group (4 cases vs. 0), representing a statistically significant difference (P = 0.033). Other symptoms, including dyspnea, fatigue, weight loss, and hepatosplenomegaly, were observed in both groups but without significant intergroup variation. Anemia was present in all cases.

**Table 2:** Clinical symptoms.

Symptoms	Total(N=19)	Non-disseminated TB(N=9)	Disseminated TB(N=10)	χ²	P value
Cough	12	7	5	1.571	0.210
Expectoration	12	7	5	1.571	0.210
Blood in phlegm or hemoptysis	1	1	0	1.173	0.279
Chest pain	1	1	0	1.173	0.279
Dyspnea tachypnea	5	2	3	0.148	0.701
fever	18	8	10	1.173	0.279
Night sweats fatigue	1	0	1	0.950	0.330
Weak	13	5	8	1.310	0.252
Weight loss	14	5	9	2.898	0.089
Nausea and vomiting	1	1	0	1.173	0.279
Abdominal pain and diarrhea	3	2	1	0.532	0.466
hepatosplenomegaly	13	6	7	0.024	0.876
Central nervous system symptoms	4	0	4	4.560	0.033
Rash	1	0	1	0.950	0.330
Skin ulcer	2	0	2	2.012	0.156
Lymphadenectasis	8	4	4	0.038	0.845
Anemia	19	9	10	/	/

### 3.3 Diagnosis of hemophagocytic lymphohistiocytosis

As shown in **Table 3**, there were no statistically significant differences in the incidence of HLH diagnostic criteria between the two groups (all P-values > 0.05). Fever (>38.5 °C), hemoglobin reduction (<90 g/L), and the presence of hemophagocytosis on bone marrow aspirate were each present in 18 of 19 patients, with similar distributions between groups. Elevated ferritin (≥500 µg/L) and splenomegaly were also commonly observed. Although thrombocytopenia, hypertriglyceridemia, and hypofibrinogenemia were slightly more frequent in the disseminated TB group, but differences did not reach statistical significance.

**Table 3:** Diagnostic-criteria for hemophagocytic syndrome.

Diagnostic criteria	Total(N=19)	Non-disseminated TB(N=9)	Disseminated TB(N=10)	χ²	P value
Fever > 38.5°C	18	8	10	1.173	0.279
Ferritin ≥ 500 µg/L	16	8	8	0.281	0.596
Elevated soluble CD25/soluble interleukin-2 receptor	0	0	0	/	/
Decreased NK-cell activity	0	0	0	/	/
Splenomegaly	12	6	6	0.090	0.764
Hemoglobin < 90 g/L	18	8	10	1.173	0.279
Hemophagocytosis visible on bone-marrow smear	18	8	10	1.173	0.279
Platelet count < 100 × 10^9^/L	11	7	4	2.773	0.096
Fibrinogen < 1.5 g/L	2	2	0	2.484	0.115
Triglycerides > 3 mmol/L	3	0	3	3.206	0.073
Absolute neutrophil count < 1.0 × 10^9^/L	1	1	0	1.173	0.279
Hepatic injury	13	6	7	0.024	0.876
Renal impairment	5	2	3	0.148	0.701
Malnutrition	18	8	10	1.173	0.279
Respiratory failure	4	3	1	1.552	0.213

### 3.4 Laboratory findings

As detailed in **Table 4**, comparison of hematologic and biochemical parameters revealed that C-reactive protein (CRP) levels differed significantly between groups. CRP was significantly lower in the disseminated TB group (73.20 ± 42.38 mg/L) than in the non-disseminated group (125.41 ± 61.45 mg/L, P = 0.044). Other key parameters, including lactate dehydrogenase (LDH), aspartate aminotransferase (AST), alanine aminotransferase (ALT), serum ferritin, albumin, and various blood cell counts (white blood cells, red blood cells, hemoglobin, platelets, neutrophils, lymphocytes), showed no statistically significant differences (P > 0.05). Notably, LDH levels in the disseminated group (288.00 ± 156.65 U/L) exhibited lower than those in the non-disseminated group (1072.22 ± 1346.25 U/L).

**Table 4:** Laboratory examination.

Hematological parameters	Total(N=19)	Non-disseminated TB(N=9)	Disseminated TB(N=10)	T value	P value
White blood cell count (WBC) (×10^9^/L)	5.83 ± 6.12	7.05 ± 8.57	4.73 ± 2.59	-0.816	0.426
Red blood cell count (RBC) (×10¹²/L)	2.50 ± 0.67	2.43 ± 0.92	2.57 ± 0.37	0.430	0.676
Hemoglobin (Hb) (g/L)	71.26 ± 16.96	69.56 ± 24.13	72.80 ± 7.21	0.388	0.707
Platelet count (PLT) (×10^9^/L)	90.74 ± 60.59	67.56 ± 53.57	111.60 ± 61.41	1.657	0.116
Neutrophil count (NEUT) (×10^9^/L)	4.69 ± 5.93	5.63 ± 8.32	3.85 ± 2.64	-0.644	0.528
Lymphocyte count (LYMPH) (×10^9^/L)	0.73 ± 0.44	0.90 ± 0.50	0.58 ± 0.33	-1.659	0.115
Monocyte count (MONO) (×10^9^/L)	0.47 ± 0.49	0.50 ± 0.36	0.45 ± 0.60	-0.216	0.831
Eosinophil count (EO) (×10^9^/L)	0.11 ± 0.34	0.03 ± 0.07	0.18 ± 0.47	0.964	0.349
Basophil count (BASO) (×10^9^/L)	0.02 ± 0.05	0.04 ± 0.07	0.01 ± 0.01	-1.296	0.230
C-reactive protein (CRP) (mg/L)	97.93 ± 57.39	125.41 ± 61.45	73.20 ± 42.38	-2.176	0.044
Aspartate aminotransferase (AST) (U/L)	94.58 ± 138.44	126.11 ± 178.81	66.20 ± 89.55	-0.939	0.361
Alanine aminotransferase (ALT) (U/L)	53.05 ± 54.11	64.67 ± 51.47	42.60 ± 56.95	-0.882	0.390
Fibrinogen (FIB) (g/L)	3.68 ± 2.00	3.68 ± 2.62	3.68 ± 1.37	-0.003	0.998
Serum iron (Fe) (μg/L)	1129.48 ± 929.99	1450.04 ± 1259.39	840.98 ± 353.11	-1.402	0.194
Lactate dehydrogenase (LDH) (U/L)	659.47 ± 989.76	1072.22 ± 1346.25	288.00 ± 156.65	-1.737	0.120
Alkaline phosphatase (ALP) (U/L)	207.06 ± 209.04	211.67 ± 204.69	202.92 ± 223.86	-0.089	0.930
Total bilirubin (TBil) (μmol/L)	29.21 ± 27.76	33.93 ± 33.04	24.97 ± 23.00	-0.693	0.498
Indirect bilirubin (IBil) (μmol/L)	10.85 ± 9.72	10.38 ± 7.27	11.27 ± 11.89	0.194	0.849
Serum creatinine (Scr) (μmol/L)	91.92 ± 77.67	105.64 ± 108.42	79.56 ± 35.48	-0.721	0.481
Albumin (ALB) (g/L)	29.95 ± 6.75	28.77 ± 6.98	31.02 ± 6.72	0.717	0.483
Serum potassium (K^+^) (mmol/L)	3.71 ± 0.39	3.65 ± 0.33	3.76 ± 0.44	0.619	0.544
Serum sodium (Na^+^) (mmol/L)	134.11 ± 5.28	135.78 ± 3.63	132.60 ± 6.22	-1.338	0.198

### 3.5 Imaging findings

**Figure 1:**
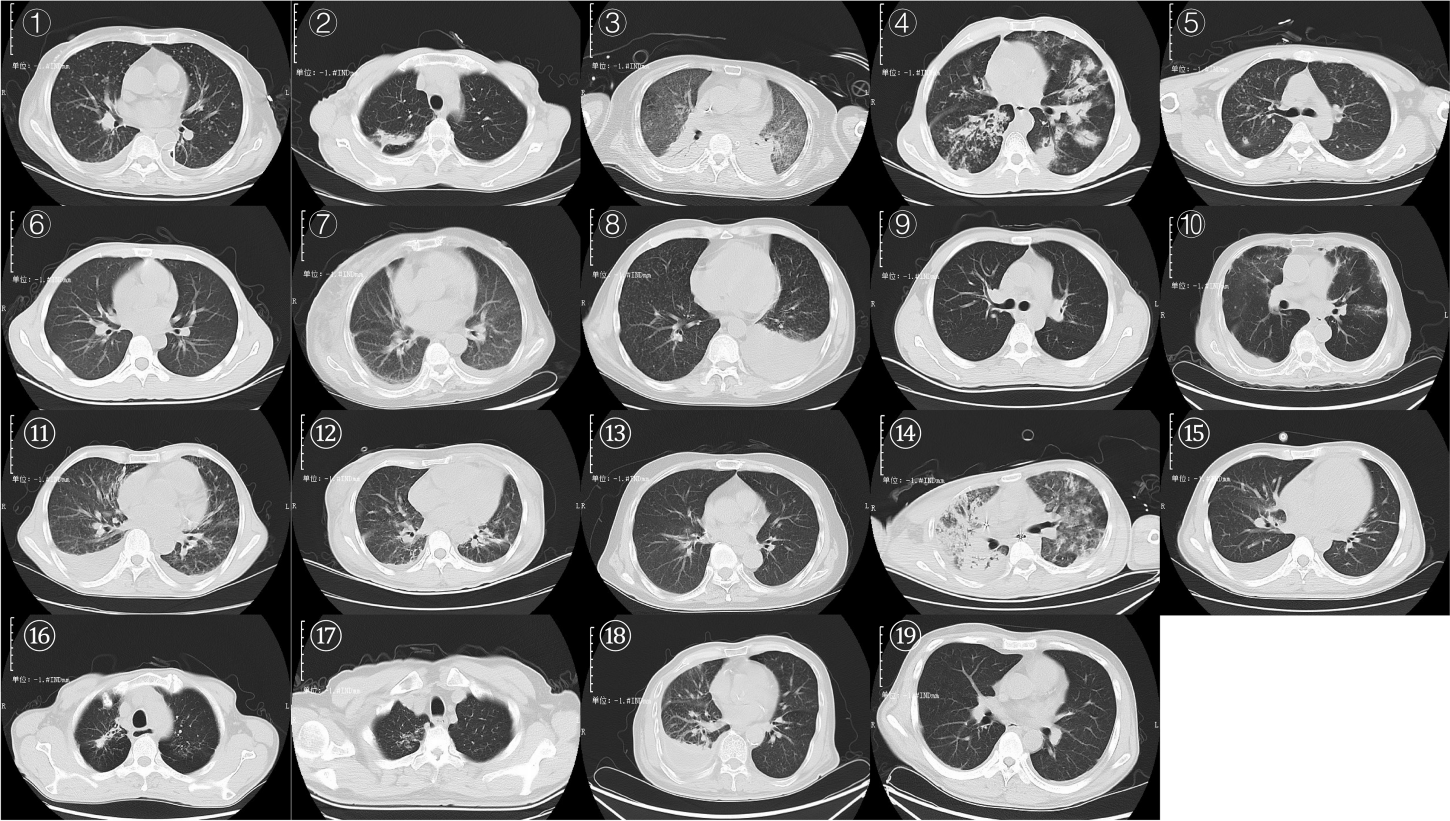
Chest CT imaging examinations of 19 patients.

Chest CT findings, summarized in **Figure 1**, showed that most patients exhibited typical radiologic features of pulmonary TB, including cavitary pneumonia, bronchiectasis, and pleural effusion. Notably, in 7 patients, imaging was described nonspecifically as “pulmonary infection” without characteristic TB features, suggesting that radiological manifestations of TB in the context of HLH may be atypical, complicating diagnosis. Additionally, 6 patients showed clinically significant extrapulmonary abnormalities, including intracranial lesions (e.g., hypodensities and infectious foci), abdominal and cervical lymphadenopathy, and involvement of ribs and pericardium. These findings supported the diagnosis of disseminated TB in several patients, emphasizing the importance of recognizing multi-system involvement.

### 3.6 Treatment and outcomes

Among all 19 patients, 14 (73.7%) showed clinical improvement after treatment, 4 (21.1%) died, and 1 patient (Case 11) was discharged against medical advice due to critical illness. A total of 16 patients received glucocorticoid therapy (including prednisone, methylprednisolone, or dexamethasone), 1 case also received cyclosporine. Only 3 patients did not receive steroids: Case 2 (short disease duration, non-disseminated TB with mild disease and bone marrow suppression), Case 14 (acute hepatic and renal failure with rapid progression), and Case 17 (absence of fever, spontaneous hematological recovery after anti-TB treatment). Excluding Case 11, all patients received anti-TB regimens, primarily based on isoniazid, rifamycin derivatives, ethambutol, and fluoroquinolones. First-line drugs were prioritized, however, 4 patients had pre-treatment leukocyte counts <2.0×10^9^/L and/or platelet counts <50×10^9^/L, leading to avoidance of rifampin. Three patients who were intolerant to rifampin were switched to rifapentine. Pyrazinamide was excluded in 15 patients due to intolerance. Some patients received individualized regimens, including empirical second-line drugs (e.g., fluoroquinolones), adjusted based on drug susceptibility testing and bone marrow recovery. Adverse drug reactions occurred in five patients and included hepatotoxicity induced by rifampin and/or pyrazinamide, QTc prolongation associated with fluoroquinolones, leukopenia due to rifampin, and rifampin-related allergic reactions. Of the 4 patients who died (Cases 5, 12, 14, and 19), three (Cases 5, 12, and 19) were HIV-positive and died from severe infections and septic shock. The remaining patient (Case 14) died from acute liver and kidney failure complicated by septic shock (**Table 5**).

**Table 5:** Treatment regimens and outcomes of 19 patients.

ID	Tuberculosis site	Method of tuberculosis diagnosis	Hemophagocytic syndrome treatment regimen	Anti-tuberculosis regimen	Adverse drug reactions during current treatment	Outcome
1	Bilateral lungs	Culture	Prednisone	HRELfxLzdCfz	QTc prolongation + renal impairment	Improved
2	Pleura	Clinical diagnosis	None	HRELfx	None	Improved
3	Intracranial + lungs	AFB stain + Xpert	Cyclosporine + methotrexate + prednisone	HREZMfx	None	Improved
4	Lungs	AFB stain + Culture	Prednisone	HEMfx	Hepatic impairment induced by pyrazinamide	Improved
5	Intracranial + lungs	Clinical diagnosis	Methylprednisolone sodium succinate	HRE	None	Death
6	Blood + bone marrow	Culture	Prednisone	HEZLfx	None	Improved
7	Lungs + intracranial + lymph nodes	NGS	Prednisone	HRZLfx	None	Improved
8	Lungs	NGS	Prednisone	HRftLfx	None	Improved
9	Lungs	TB-DNA	Dexamethasone	HREZ	None	Improved
10	Lungs + intracranial + lymph nodes	NGS	Prednisone	HRftMfx	Hepatic impairment caused by rifampicin and pyrazinamide	Improved
11	Lungs	Clinical diagnosis	Methylprednisolone sodium succinate+Dexamethasone		None	Improved
12	Lungs	Clinical diagnosis	Prednisone	HREZ	None	Death
13	Blood + bone marrow + lungs + intracranial	NGS	Prednisone	HREMfx	None	Improved
14	Lungs	TB-DNA	None	HRLfxLzd	Acute hepato-renal failure	Death
15	Lungs	Clinical diagnosis	Prednisone	HRELfx	None	Improved
16	Lungs + lymph nodes	Culture + Xpert	Prednisone	HRELfx	Leukopenia	Improved
17	Lungs	Culture	None	HRftE	None	Improved
18	Lungs	Xpert	Dexamethasone	AmHEMfx	None	Improved
19	Lungs	Clinical diagnosis	Dexamethasone	AmHEMfx	None	Death

## 4 Discussion

TB-HLH is a critical clinical syndrome at the intersection of infectious diseases, hematology, and critical care medicine. It represents a dysregulated and life-threatening systemic inflammatory response triggered by MTB infection. Against the persistently high global burden of TB, TB-HLH stands out as a severe complication, with reported case fatality rate reaching up to 40% (11). The core pathophysiological mechanism of TB-HLH involves the disruption of immune homeostasis by MTB infection. Impaired cytotoxic function of T cells and NK cells leads to ineffective antigen clearance, thereby resulting in sustained immune activation. Persistent stimulation by MTB antigens drives excessive activation of lymphocytes and macrophages, which secrete large quantities of pro-inflammatory cytokines, including IFN-γ, TNF-α, and IL-6, culminating in a cytokine storm (12–14). Furthermore, MTB exploits interleukin-12 (IL-12) and interleukin-15 (IL-15) to promote the migration of monocytes and macrophages to regional lymph nodes, enhance antigen-specific T cell proliferation, sustain cytokine release, and further amplify macrophage activation and expansion (15–17). This process not only causes direct tissue and organ damage but also leads to aberrant activation of macrophages in hematopoietic tissues like the bone marrow and spleen, resulting in hemophagocytosis and the characteristic clinical triad of pancytopenia, fever, and hepatosplenomegaly (18). There is substantial overlap in the clinical presentations of TB and HLH, including fever, hepatosplenomegaly, pancytopenia, and elevated inflammatory markers. This similarity complicates the recognition of HLH in patients with active TB, as signs of HLH are often masked by the systemic inflammatory response associated with TB itself. Non-specific symptoms, such as persistent fever, hepatosplenomegaly, and cytopenias (particularly thrombocytopenia), frequently lead clinicians to prioritize more common differential diagnoses, including malignancies, other infections, metabolic disorders, or rheumatic diseases, thereby increasing the diagnostic challenge for TB-HLH (6). This study confirms that fever, hepatosplenomegaly, and cytopenias are nearly universal core clinical features of TB-HLH, consistent with classical descriptions of HLH.

In this study, all patients underwent bone marrow aspiration to confirm the diagnosis of HLH. Among them, hemophagocytosis was observed in 18 cases (94.7%), providing direct pathological evidence for their cytopenia. In HLH, the etiology of cytopenia is complex and results from the interplay of multiple mechanisms. The primary mechanism involves active hemophagocytosis within the bone marrow, where activated macrophages directly engulf hematopoietic cells, leading to impaired effective hematopoiesis (19). Secondly, the cytokine storm associated with HLH (such as elevated levels of IFN-γ) can trigger immune-mediated peripheral blood cell destruction, while concurrent splenomegaly often leads to hypersplenism, accelerating the sequestration and clearance of blood cells (20, 21). Furthermore, chronic tuberculosis infection and systemic hyperinflammatory states can directly suppress bone marrow hematopoiesis through inflammatory cytokines. When combined with malnutrition, this may further exacerbate bone marrow suppression. Therefore, the evaluation of cytopenia in TB-HLH patients requires a comprehensive assessment that integrates bone marrow morphology, cytokine levels, and systemic inflammatory status.

In terms of clinical manifestations, while fever is a common symptom of active tuberculosis, significant differences exist between TB-HLH patients and those with simple tuberculosis. This study demonstrates that persistent high fever (body temperature >38.5 °C lasting over seven days) is almost a universal feature among TB-HLH patients. Such high fever often shows poor responsiveness during the initial phase of anti-tuberculosis treatment, presenting as refractory. In contrast, fever in patients with simple pulmonary tuberculosis is generally milder and shorter in duration, typically resolving rapidly upon receiving effective anti-tuberculosis therapy (22). Furthermore, data from this study indicate that the incidence of hepatosplenomegaly is significantly higher in TB-HLH patients compared to those with general active pulmonary tuberculosis. In cases of simple tuberculosis, marked hepatosplenomegaly is uncommon unless accompanied by abdominal tuberculosis or severe malnutrition (23). This difference in clinical presentation further suggests that TB-HLH may involve more extensive systemic involvement and a more intense state of immune activation, with hepatosplenomegaly serving as an important clinical indicator of progression from tuberculosis infection to hemophagocytic syndrome. In this study, 40% of patients in the disseminated TB-HLH group exhibited CNS symptoms (e.g., headache, impaired consciousness), and a small number of patients presented with rash (5.3%) and skin ulcers (10.5%). In contrast, such symptoms are extremely rare in patients with simple pulmonary tuberculosis (without dissemination). Regarding laboratory indicators, this study observed a noteworthy finding: serum CRP levels were significantly lower in patients with disseminated TB-HLH compared to those with non-disseminated TB-HLH. Generally, the severity of infection or inflammation correlates positively with CRP levels, meaning that more severe conditions lead to higher CRP elevation. For instance, in cases of simple disseminated tuberculosis (such as miliary tuberculosis), CRP typically shows a marked increase (24). A similar paradoxical pattern was noted for LDH. Although the difference between groups did not reach statistical significance, LDH levels in the disseminated TB-HLH group were considerably lower than those in the non-disseminated group. Additionally, this study observed that compared to patients with simple tuberculosis, TB-HLH patients exhibited more pronounced abnormalities in several laboratory indicators, including ferritin, coagulation function, and lipid profiles.

Research indicates that miliary tuberculosis, a fatal disseminated form of the disease, may be associated with an insufficient effector T−cell immune response, in which chemokine−mediated selective homing of Th2 cells may play a critical role. In contrast, non−disseminated tuberculosis lesions are mostly confined to the lungs or local tissues, with a relatively localized inflammatory response and a lower degree of systemic immune activation and impact. Clinically, miliary tuberculosis can be accompanied by various rare and severe complications. Cases characterized by acute respiratory distress syndrome, air−leak syndrome, acute kidney injury, and hepatic or gastrointestinal damage are increasingly reported in emergency departments and intensive care units. These manifestations may either be part of a tuberculosis−associated multiple organ dysfunction syndrome or related to immune reconstitution inflammatory syndrome. Given the significant differences in immunological mechanisms and clinical presentations between the two forms, this study aims to conduct a systematic comparative analysis of disseminated (miliary) pulmonary tuberculosis and non−disseminated pulmonary tuberculosis.

Additionally, symptoms indicative of CNS involvement showed significant specificity for disseminated TB (P = 0.033). All four patients presenting with headache, dizziness, seizures, or impaired consciousness exclusively belonged to the disseminated TB group. This finding has crucial clinical implications. In practice, the presence of neurological symptoms in patients with TB-HLH should raise strong suspicion of hematogenous dissemination of MTB and warrant immediate neurological evaluation, including contrast-enhanced cranial MRI and cerebrospinal fluid analysis, to assess for intracranial TB manifestations such as tuberculous meningitis or tuberculomas. Early recognition is crucial not only for accurate disease staging but also for optimizing anti-tuberculosis therapy, such as deciding whether to incorporate agents with good blood-brain barrier penetration, like pyrazinamide, levofloxacin, or linezolid (25, 26).

A counterintuitive and clinically intriguing observation in this study was that patients with disseminated TB-HLH had significantly lower CRP levels than those with non-disseminated disease (73.20 ± 42.38 mg/L vs. 125.41 ± 61.45 mg/L, P = 0.044). CRP is a classical acute-phase inflammatory marker that typically correlates positively with the severity of infection or inflammation, with higher levels conventionally indicating more severe disease (24). One plausible explanation is that disseminated TB, through extensive tissue involvement and prolonged antigenic stimulation, may induce immune exhaustion. This state of immune dysfunction could impair hepatic synthesis of acute-phase proteins, leading to a relative reduction in CRP production despite severe systemic illness (27). This finding suggests that in TB-HLH, particularly in suspected disseminated cases, an unexpectedly low CRP level that is disproportionate to the clinical severity may itself represent a distinctive immunological phenotype, potentially reflecting immune exhaustion or a specific cytokine storm profile. Concurrently, although not statistically significant, LDH levels showed a markedly lower trend in the disseminated group (288.00 ± 156.65 U/L vs. 1072.22 ± 1346.25 U/L). This observation may indicate differences in the pattern or extent of cellular damage and warrants further investigation in larger cohorts.

The cornerstone of TB-HLH management lies in rapidly controlling the source of infection with anti-tuberculosis therapy (ATT) while simultaneously suppressing the hyperinflammatory immune response through immunomodulatory or immunosuppressive strategies. Most ATT regimens are built around isoniazid (H) and ethambutol (E) as foundational agents (6, 9). In cases with severe bone marrow suppression (e.g., white blood cell count <2.0×10^9^/L and/or platelet count <50×10^9^/L) or intolerance to rifamycins, fluoroquinolones such as levofloxacin (Lfx) or moxifloxacin (Mfx) are often substituted or incorporated into intensive regimens (e.g., H-E-Lfx regimen) (28). In refractory or complex cases, additional agents such as linezolid (Lzd), clofazimine (Cfz), or amikacin (Am) may be included to optimize efficacy while minimizing hematologic toxicity. In this study, five patients (26.3%) experienced adverse drug reactions, including myelosuppression, hepatotoxicity, QTc prolongation, and allergic reactions, highlighting the altered drug metabolism and tolerance often seen in HLH and underscoring the need for close monitoring throughout treatment (29). Immunomodulatory therapy, particularly glucocorticoids, plays a pivotal role in the management of infection-associated HLH. In our cohort, 84.2% (16/19) received glucocorticoids, aligning with international consensus recommendations advocating early steroid use in secondary HLH (9). However, the use of immunosuppressive agents introduces a critical therapeutic paradox, while they suppress the cytokine storm, they may also exacerbate underlying MTB infection (6). Corticosteroids non-specifically suppress lymphocyte activation and cytokine release, rapidly leading to rapid clinical stabilization. The three patients who did not receive steroids had distinct clinical scenarios: Case 2 had a short disease duration, mild illness bone marrow suppression, Case 14 bone marrow suppression multi-organ failure, and Case 17 achieved spontaneous hematological recovery and remained afebrile with ATT alone. Case 3 n intensive immunosuppressive regimen comprising prednisone, cyclosporine, and methotrexate, similar to protocols used for primary or refractory HLH, to control a fulminant cytokine storm (30). Among the fatal cases, three patients had concurrent HIV infection and died of severe infection with septic shock. The overall mortality rate in this study was 21.1% (4/19). Detailed analysis identified two principal high-risk factors: (1) Underlying immunodeficiency: HIV-induced depletion of CD4+ T cells predisposes to disseminated TB and disrupts normal immune regulation, increasing susceptibility to HLH and opportunistic infections. (2) Specific organ failure: Case 14 died from acute hepatic and renal failure, both hallmark complications of cytokine storm–mediated end-organ damage. These failures impair drug metabolism and clearance, potentially exacerbating the inflammatory response and therapeutic toxicity. These factors emphasize the importance of rapid risk stratification in TB-HLH patients. Clinical indicators such as AIDS, CNS symptoms, rapidly progressive multiple organ dysfunction (particularly hepatic and renal failure), and paradoxical laboratory findings suggestive of immune exhaustion (such as relatively low CRP levels) should be regarded as high-risk markers. Such patients may benefit from early intensive care admission and aggressive multidisciplinary intervention.

This study preliminarily delineates the characteristic profile of TB-HLH across different spectrums of tuberculosis, providing insights for more refined clinical research and diagnostic-therapeutic strategies in the future, yet it still has certain limitations. As a single-center retrospective analysis with a small sample size, statistical power may be limited, particularly in subgroup comparisons. Furthermore, the absence of long-term follow-up data precludes evaluation of long-term relapse rates, treatment-related complications, and survival outcomes beyond hospital discharge. Future research should focus on establishing multicenter, prospective registries to enroll larger patient cohorts and enable collection of comprehensive immunological data, including cytokine profiles, lymphocyte subsets, sCD25 levels. Additionally, prospective studies employing risk-stratified treatment strategies are needed to identify which patients would derive the most benefit from early immunomodulatory therapy, and to determine optimal therapeutic windows, drug combinations, and dosing strategies tailored to disease severity.

## Data Availability

The original contributions presented in the study are included in the article/supplementary material. Further inquiries can be directed to the corresponding authors.
